# Distribution and Driving Factors of Forest Swamp Conversions in a Cold Temperate Region

**DOI:** 10.3390/ijerph15102103

**Published:** 2018-09-25

**Authors:** Dandan Zhao, Hong S. He, Wen J. Wang, Jiping Liu, Haibo Du, Miaomiao Wu, Xinyuan Tan

**Affiliations:** 1 School of Geographical Sciences, Northeast Normal University, Changchun 130024, China; zhaodd982@nenu.edu.cn (D.Z.); duhb655@nenu.edu.cn (H.D.); wumm836@nenu.edu.cn (M.W.); tanxy315@nenu.edu.cn (X.T.); 2 School of Natural Resources, University of Missouri, Columbia, MO 65211, USA; 3 Northeast Institute of Geography and Agroecology, Chinese Academy of Sciences, Changchun 130102, China; wangwenj@iga.ac.cn; 4 School of Tourism and Geography Science, Jilin Normal University, Siping 136000, China; liujpjl@163.com

**Keywords:** forest swamps, swamp conversions, variable importance measure, cold temperate regions

## Abstract

Forest swamps are widely distributed in cold temperate regions, with important landscape and ecological functions. They are prone to conversion caused by complex factors. Forest swamp conversions involve forest swamping, meadow swamping, water body swamping, and conversion to farmland. An understanding of the landscape characteristics and primary environmental factors driving forest swamp conversions is imperative for exploring the mechanism of forest swamp conversions. We investigated the landscape characteristics of forest swamp conversions and quantified the relative importance of environmental factors driving these conversions for the period from 1990 to 2015 in the Great Xing’an Mountains of China. We found that forest swamping displayed high patch numbers (34,916) and density (8.51/100 ha), commonly occurring at the edge of large areas of forests. Meadow swamping was localized with low patch numbers (3613) and density (0.88/100 ha) due to lack of water recharge from ground water. Water body swamping had complex shapes (perimeter area ratio mean = 348.32) because of water table fluctuations and helophyte growth during this conversion process. Conversions to farmland presented fairly regular (perimeter area ratio mean = 289.91) and aggregated (aggregation index = 67.82) characteristics affected by agricultural irrigation and management. We found that climatic and geomorphic factors were relatively important compared to topographic factors for forest swamp conversions. Negative geomorphic conditions provided the waterlogging environment as a precondition of swamp formation. Sufficient precipitation was an important source of water recharge due to the existence of permafrost regions and long-term low temperature reduced the evaporation of swamps water and the decomposition rate of organisms. These wet and cold climatic conditions promoted forest swamp development in cold temperate regions. Humans exerted a relatively important role in forest swamping and conversions to farmland. Fire disturbance and logging accelerated the conversion from forest to swamp. This study provides scientific information necessary for the management and conservation of forest swamp resources in cold temperate regions.

## 1. Introduction

Forest swamps are hygrophyte and helophyte communities formed through long-term low temperature and perennial waterlogging [1]. They are widely distributed in north temperate regions, with enormous ecological functions [2,3]. Forest swamps regulate the regional climate, affecting the carbon sequestration and oxygen in the environment through plant respiration and photosynthesis [4,5], which are of significance to the carbon and oxygen balance of ecosystems [6]. Forest swamps have crucial functions in water conservation, such as water storage and purification, runoff regulation, and flood interception [6,7,8]. Forest swamps also maintain species diversity acting as the intersections between forests and swamps [9,10].

Forest swamps can be formed in virtue of forest swamping, meadow swamping, and water body swamping [11]. Forest swamping refers to the conversion from forest to swamp with the spread of hygrophytes and helophytes caused by moist soil or water accumulation because the disturbance degrades forest transpiration capacity and forest dieback may also decrease transpiration resulting in excessive accumulation of water [12,13], which is difficult to infiltrate due to the presence of permafrost layers and low temperature conditions [11,14,15]. Meadow swamping means the conversion from meadow to swamp, with increases in peat accumulation because the organic plant residues cannot be fully mineralized and decomposed under conditions of excessive moisture, high groundwater level, anaerobic environment, and poor microbial activities [16,17]. Water body swamping is the conversion from water bodies (rivers and lakes) to swamp through mechanical, chemical, and biological depositions [11,18]. Meanwhile, aquatic plants begin to appear around the shores and the submerged plant residues accumulate year by year due to slow decomposition in the oxygen-poor environment that cause helophytes invading toward deep water level to form the swamps [19,20]. 

On the country, the conversion from forest swamps to farmland generally denotes that humans convert forest swamps into farmland through drainage irrigation due to food and economic needs and related polices [21,22,23]. Conversions of forest swamp to or from other land types are often driven by complex environmental factors [15,24,25]. Under what circumstances a given conversion may occur is of great interest for understanding the landscape characteristic of change and the potential driving factors, especially under a changing climate. Thus, research on the effects of complex environmental factors on forest swamping, meadow swamping, water body swamping, and conversions to farmland can provide valuable insights into current forest swamp management decisions and conservation strategies.

The Great Xing’an Mountains of Northeastern China contain rich forest swamps in a cold temperate forested region [26]. This forested region has experienced forest swamping, meadow swamping, water body swamping, and conversions to farmland. Complex environmental factors, such as climatic, topographic, geomorphic, edaphic, hydrologic, and human influences in recent decades are believed to have played a significant role in swamp conversion [11,25,27]. However, distribution and conversion processes of forest swamps remain unclear due to the uncertainty of the relative importance of environmental factors. 

In our study, we investigated forest swamping, meadow swamping, water body swamping, and conversions to farmland as they were the predominant conversion types. We investigated two specific issues. Firstly, what were the landscape characteristics of forest swamp conversions? Secondly, what was the relative importance of environmental factors that affected forest swamp conversions in this cold temperate region?

## 2. Materials and Methods

### 2.1. Study Area

**Figure 1:**
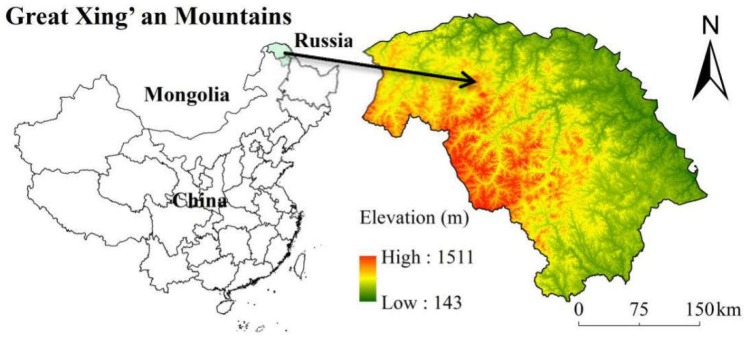
Study area: the Great Xing’an Mountains in Northeast China.

The Great Xing’an Mountains (50°–53° N and 121°–127° E) are located in Northeast China at the border with Russia, and represent a cold temperate region with an area of 8,040,437 ha (Figure 1). The elevation ranges from 143 to 1511 m above sea level and there is a cold temperate continental monsoon climate, with mean annual temperature from −6 to 1 °C and mean annual precipitation from 240 to 442 mm. Spring and autumn is dry and windy whereas the winter is cold and long affected by Mongolian and Siberian high pressure. The Great Xing’an Mountains are formed by as much as 75–80% of forest, representing an important forested base in China. The study area contains abundant swamps in this forested region. Previous studies on forest swamp conversions have concentrated on the middle temperate region and warm temperate region of the northern temperate region and less attention has been paid to cold temperate regions.

### 2.2. Study Object

Forest swamps refer to the vegetation in the habitat with excessive soil moisture, accumulation of water or shallow water, and peat in the forested region. The vertical structure of plant communities in the study area is composed of four levels, including two main tree species, *Larix gmelinii* (Rupr.) Kuzen. and *Betula platyphylla* Sukaczev; five main important shrubs, *Betula fruticosa* Pall., *Rhododendron parvifolium* Adams, *Ledum palustre var. angustum* E.A. Busch, *Vaccinium uliginosum* L., and *Salix rosmarinifolia* L.; four representative herbs, *Eriophorum vaginatum* L., *Carex globularis* L., *Carex schmidtii* Meinsh., and *Carex rhynchophysa* Fisch., C.A.Mey. & Avé-Lall.; and four dominant mosses, *Sphagnum magellanicum* Brid., *Sphagnum fuscum* (Schimp.) H. Klinggr., *Polytrichum juniperinum* Hedw., and *Ptilium crista-castrensis* (Hedw.) De Not. [28,29,30]. The vegetation is almost hygrophilous and cold-resistant. *Larix gmelinii* (Rupr.) Kuzen. is a light-demanding species with high water requirements, which can adapt to various environmental conditions (e.g., mountains, swamps, peatland, meadows, wet soil, and wet river valleys) [31]. *Betula platyphylla* Sukaczev is a light-demanding tree growing in acidic soils, and can grow in swamps, dry sunny slopes, and wet shady slopes [32]. *Betula fruticosa* Pall. mainly grows in the swamp or along the river [33,34]. *Rhododendron parvifolium* Adams is generally found in the alpine grassland, shrub, or mixed forest [35]. *Ledum palustre var. angustum* E.A. Busch has strong cold resistance and is mostly found in the mountain peat moss swamps or alpine meadow swamps [36]. *Vaccinium uliginosum* L. is resistant to acid soil environment, low temperature, and drought [37]. *Salix rosmarinifolia* L. is mainly grown in forested regions or swampy meadows [38]. Herbs are common in the hillside grassland, swamp, and along the lakes and rivers [39,40,41] and mosses are generally found in mountainous wet areas and swamps [42,43,44]. 

### 2.3. Land Use Land Cover Datasets

Remote sensing images of land use land cover (LULC) can help us to characterize the changes on the land surface through the use of time series data [45]. This advantage makes remote sensing become the best means to identify the forest swamp conversions. We used cloud-free Landsat Thematic Mapper (TM) and Enhanced Thematic Mapper Plus (ETM+) images with a spatial resolution of 30 m in 1990 and 2015 to classify LULC of the Great Xing’an Mountains. These remote sensing images were downloaded from the USGS Center for Earth Resources Observation and Science (http://glovis.usgs.gov). In order to ensure the consistency of images acquisition time, the interpretation process of remote sensing was based on the images with the largest biomass season (July and August). We corrected them using radiometric calibration [46] and FlAASH atmospheric correction models [47] to eliminate radiometric and atmospheric effects by ENVI 5.2. We performed the classification using an image-processing program of object-based information analysis with eCognition Developer 8.64 software (Trimble Navigation Limited, Sunnyvale, CA, USA) [45,48]. First, we used a multi-resolution segmentation method to segment the images into homogeneous objects according to scale, shape, and compactness [49]. Second, we used classification rule sets to classify LULC based on the hierarchical classification method automatically and the classification rules were closely related to the characteristics (e.g., brightness, size, and adjacency) of an object [50,51]. We used the normalized differential vegetation index (NDVI) to identify the vegetated areas from non-vegetated areas after the primary segmentation. We selected the ratio vegetation index (RVI), the normalized difference water index (NDWI), the bright index, the hue index, and the length/width to establish hierarchical classification for distinguishing LULC. These indexes were derived from images and the specific classification threshold referred to previous studies [52,53,54]. Third, we revised the preliminary classification results through visual interpretation [55] and overlaying the classification results and assistant data (vegetation type, the time of year covered by water (month), and humidity index) [56]. The vegetation type was derived from the 1:1 million Chinese vegetation maps, the time of year covered by water (month) was derived from images, and humidity index was calculated through the average monthly temperature and precipitation based on the climatic data [57]. Finally, we classified LULC into six types, including forests, meadows, farmland, water bodies (rivers, lakes, and reservoirs), forest swamp, and other lands. Studies have demonstrated that the objected-oriented classification method had good ability for LULC classification [45,51,58]. We validated the classification result in 1990 by comparing with previous studies [59,60] that confirmed the classification accuracy in 1990 was above 80%. We validated classification results in 2015 using images from Google Earth, field surveys, and interviews with local people. We randomly selected 300 ground survey verification samples with a Global Positioning System (GPS) and verified the accuracy of LULC classification results with the overall classification accuracy of 85% for the year 2015. Because of the inconsistent resolutions, we uniformed the LULC classification of 1990 and 2015 into a resolution of 90 m as well as environmental factors. 

### 2.4. Environmental Factors Datasets

Forest swamp conversions are affected by complex environmental factors [11] and we selected climatic, topographic, geomorphic, edaphic, hydrologic, and human as environmental factors for forest swamp conversions including nine variables [61,62,63]. We took the mean annual temperature and mean annual precipitation as the variables of the climatic factor. Climatic data (1961–2015) came from the China Meteorological Administration and the Meteorological Data Center (http://data.cma.cn/site/index.html). We took slope and Beer’s aspect as the variables of topographic factor and we extracted slope and Beer’s aspect from the digital elevation model (DEM) with a resolution of 30 m. We took the distance to rivers as the variable of hydrologic factor due to the inaccessibility of other hydrologic data such as underground water. We took the geomorphic type as the variable of geomorphic factor and geomorphic type was extracted from the 1:4 million Chinese geomorphic maps. We took the soil type as the variable of edaphic factor and soil data was derived from 1:1 million Chinese soil maps. We took the distance to roads and human interference degree as the variables of human factor. The road map we used was in 2009 because historic road maps were not available. Human interference degree was calculated by the LULC data [64]. Because of inconsistent resolutions, we resampled the maps of environmental factors into 90 m resolution.

### 2.5. Landscape Metrics

We selected five common landscape metrics at class level to quantify landscape characteristics of forest swamp conversions, including number of patches (NP), patch density (PD), edge density (ED), perimeter area ratio mean (PARA_MN), and aggregation index (AI). We calculated the landscape metrics in the landscape pattern analysis software Fragstats 4.2 (University of Massachusetts Amherst, Amherst, MA, USA). Additionally, the meaning of the above landscape metrics in the landscape pattern and their calculation formulas are clearly described in Fragstats 4.2 [65].

### 2.6. The Variable Importance

We ranked the relative importance of environmental factors in different conversions of forest swamp by variable importance measures (VIM) in random forest (RF). RF was implemented through a Breiman random forest algorithm based on many decision trees as an ensemble learning technology [66], which can be used to explain the nonlinear and collinear relationships among environmental variables and handle a flexible number of input variables [67,68]. For each tree in the RF model, a random set of variables and a random sample from the dataset for training were selected [69]. The VIM in the RF usually adopted the importance score of permutation-based [70,71], which could be defined as the average reduction in classification accuracy of the classification model before and after a slight disturbance of the out-of-bag data [72,73]. The mean decrease in accuracy (MDA) can be considered as a means of calculating variable importance measures (VIM) [74,75]. MDA quantified variable importance through measuring the change in RF prediction accuracy, when the variable values were randomly permuted compared to original observations [76,77]. The larger MDA value denoted that the variable was more important [78]. Furthermore, we used random forest model to calculate the MDA of environmental factors for different forest swamp conversions. The RF model came from a package in R (The University of Auckland, Auckland, New Zealand) (http://www.R-project.org).

### 2.7. Data Analysis

We explored the spatial distribution of forest swamp conversions through intersecting the forest, meadow, water body, farmland, and forest swamp layers using ArcGIS 10.2 (Environmental Systems Research Institute, Redlands, CA, USA) (http://resources.arcgis.com/zh-cn/help/main/10.2/). Post classification comparison technique [79,80,81] was used to calculate the areas of forest swamping, meadow swamping, water body swamping, and conversions to farmland, respectively, by using raster comparisons. We investigated the landscape characteristics of these forest swamp conversions and quantified the relative importance of environmental factors for these conversions. 

## 3. Results

**Figure 2:**
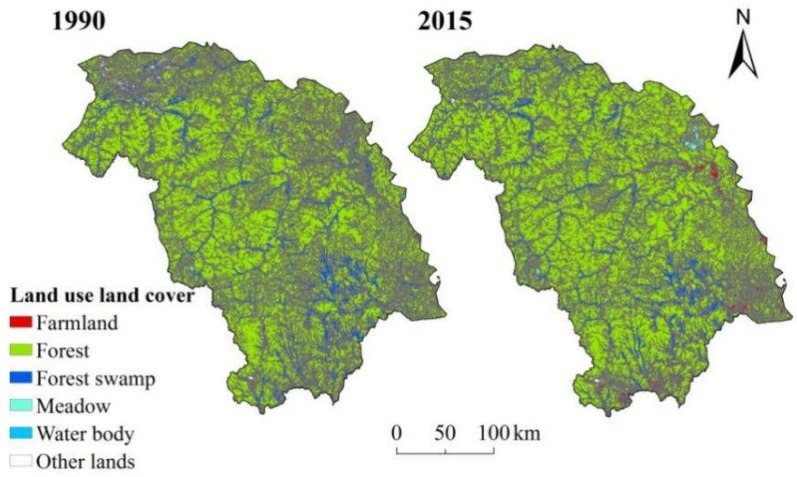
LULC during the period 1990–2015.

LULC maps showed that the study area was dominated by forest (75–80%) and forest had a slight increase of 2.46% during the period 1990–2015 (Figure 2). Meadow and forest swamp were scattered throughout the study area sporadically (Figure 2) and they reduced by 58.37% and 10.08%, respectively. Water body accounted for less than 1% of the entire study area (Figure 2), with an increase of 14.8%. Farmland increased significantly during the period 1990–2015 (Figure 2), with a total increase of 1323.83 km^2^ (265.57%).

**Figure 3:**
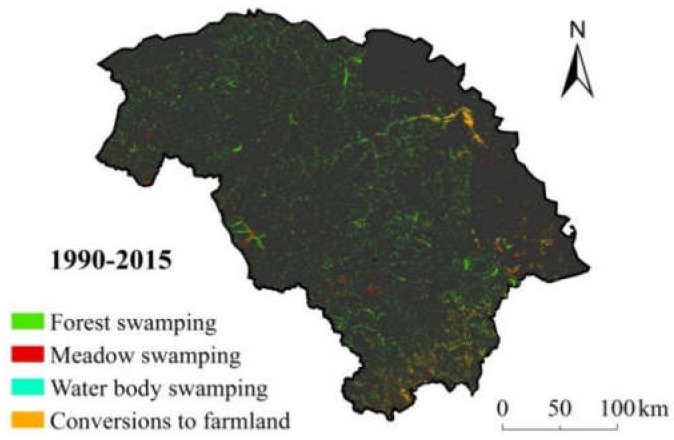
Spatial distribution of forest swamp conversions during 1990–2015.

Forest swamping was the most widespread conversion among these four forest swamp conversions (296,961 ha) and distributed throughout the study area (Figure 3). Meadow swamping presented sporadic conversion (30,368 ha) in the middle of forest swamping (Figure 3). Water body swamping generally occurred near the water body (10,143 ha) (Figure 3). Conversions to farmland involved regional conversion (66,948 ha) and were distributed in areas in the south of the study area (Figure 3).

Forest swamping was a common phenomenon (e.g., NP was 34,916 and PD was 8.51/100 ha) (Table 1). Meadow swamping had localized landscape characteristics (e.g., NP was 3613 and PD was 0.88/100 ha) (Table 1). Water body swamping had a complex shape (e.g., PARA_MEAN was 348.32) (Table 1). Conversion to farmland was a regionally aggregated conversion (e.g., AI was 67.82%) (Table 1).

The characterization of environmental factors are shown as follows (Table 2).

**Figure 4:**
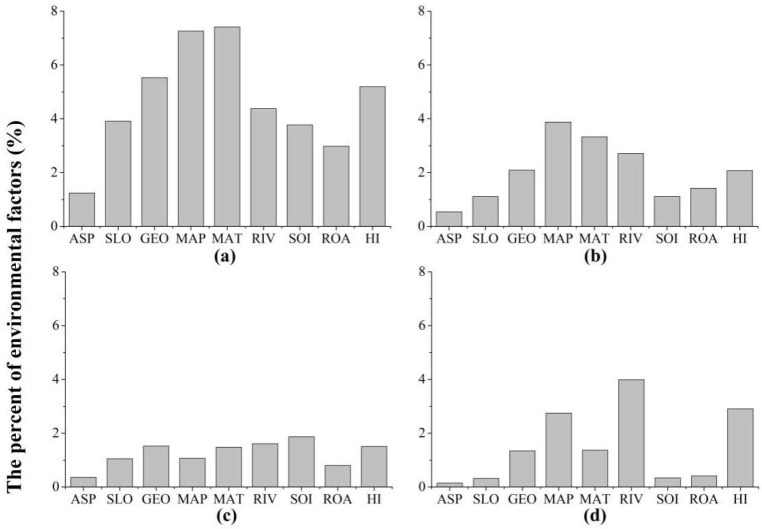
Percentages for the importance of environmental factors for different forest swamp conversions. (**a**) Forest swamping; (**b**) Meadow swamping; (**c**) Water body swamping; (**d**) Conversion to farmland. ASP: Beer’s aspect; SLO: slope; GEO: geomorphic type; MAP: mean annual precipitation; MAT: mean annual temperature; RIV: distance to rivers; SOI: soil type; ROA: distance to roads; HI: human interference degree.

On average, the mean annual temperature, mean annual precipitation, geomorphic type, and human interference degree had higher explanation power for forest swamping than other environmental variables (Figure 4). Mean annual precipitation, mean annual temperature, distance to river, and geomorphic type were the primary determinants of the meadow swamping (Figure 4). Soil types, distance to rivers, geomorphic types, and human interference degree were relatively important for the appearance of water body swamping (Figure 4). Additionally, distance to rivers, human interference degree, mean annual precipitation, and mean annual temperature were the main driving factors for conversions to farmland (Figure 4).

## 4. Discussion

Forest swamping was an important means of forest swamp formation in cold temperate regions. Dense canopy forests and the permafrost layers under the forests in cold temperate regions provided cold and wet conditions for swamp development [82,83]. Our results showed that forest swamping widely occurred under such conditions in the study area [84]. Simultaneously, forest swamping had a fairly high patch number and density and edge density, which meant this conversion presented fragmentation. The reason for this phenomenon may be that forest swamping mostly occurred at the edge rather than the core of large areas of forest [85]. Previous studies showed that topography (e.g., slope) and time elapsed since fire had important effects on the boreal forest swamping in a cold forested region of Canada [86]. Time since fire was an important factor influencing forest swamping, as the burned forests lost enormous water evapotranspiration capacity, resulting extensive soil water accumulation and dead plant residuals transforming into peat land [24]. Our results revealed climatic (e.g., mean annual temperature and mean annual precipitation), geomorphic, and human (e.g., human interference degree) factors were more important than other environmental factors in forest swamping. The reason for this difference was that our study area included a variety of geomorphic types (such as low-altitude hills, undulating mountains of varying altitudes, and low-altitude alluvial plains) rather than a single geomorphic type (e.g., lowlands). The negative basins among multiple geomorphologies could maintain a cold waterlogging environment in cold temperate regions [25,87].

Meadow swamping was a more localized phenomenon than forest swamping. Meadow swamping required long-term soil pores to be filled by surface water and ground water [88] but there was a lack of water recharge from ground water due to the abundant permafrost layers in cold temperate regions, hence our results showed that the patch number and density of meadow swamping were relatively small. Also, local changes in the groundwater table may lead to meadow swamping as a result of the silting of abandoned drainage channels [89]. Results demonstrated that climatic (e.g., mean annual precipitation and mean annual temperature), geomorphic, and hydrologic factors were the primary causes for meadow swamping. This also reconfirmed the previous studies that meadow swamping was the result of the combination of climatic, geomorphologic, and hydrological conditions in cold temperate forested region [90]. Because long periods of low temperature in cold temperate regions led to the presence of large areas of permafrost, water from river recharge and precipitation could not infiltrate [91]. Excessive water kept wet geomorphy in a state of waterlogging, promoting the growth of peat-forming plants [24,92]. The dead plant residues could not be fully decomposed under anaerobic conditions, formed into peat gradually [93], and made meadow convert to swamp. Additionally, meadow swamping was prone to occurring, as was forest swamping [11]. Our results showed that the relative importance of environmental factors on meadow swamping was similar to that for forest swamping. Therefore, further studying the conversion between specific types of forest swamps will be more conducive to exploring the succession process of forest swamps.

Water body swamping presented complex shape throughout the study area. It was likely that the area and extent of forest swamp changed through the fluctuation of water table and helophyte invasion and growth during the process of water body swamping [94]. Water body swamping was clearly different from forest swamping and meadow swamping with climatic factor predominating. Edaphic, hydrologic, and geomorphic factors were the main factors for water body swamping. These results were similar to those reported by Partanen and Luoto [19] showing that the cover of clay soil, total phosphorus content, and spring flooding were the primary determinants of the water body (e.g., lake) swamping in Finland. The reason was that when the soil experienced long-term water saturation, swamps developed with the increases of hygrophilous plants [11,95]. Accumulation of these plant death remnants formed peat due to slow decomposition under anoxic conditions, which made water bodies shallow and increased helophyte, promoting water body swamping [94]. However, our study did not take the trace elements (e.g., total phosphorus content) into account to study water body swamping. This was because pollution of water body was relatively low in the forested regions [96]. Additionally, our results showed the human factor (e.g., human interference degree) had a certain effect on water body swamping. This result differed from previous studies suggesting that water body swamping was a natural succession process in a cold temperate region [11]. The difference may be that human land use affected water body distribution, which affected water body swamping indirectly [64,97].

Areas of conversion to farmland showed not too many patches, but with simple shape and high aggregation because agricultural development converted forest swamp to paddy fields and dry lands with regular shape for convenient irrigation and management [98]. Conversion to farmland was a regional and aggregated phenomenon (e.g., the AI was the highest) in the southern part of the study area. Previous studies revealed that the large increase in regional population was the most direct and primary diving factor for the conversion from swamp to farmland in a northern temperate plain region [99]. The reason was that the increasing population reclaimed the swamp for farmland due to grain demands and economic pressure in the region [98]. Our result found that hydrologic, human, and climatic factors accounted for conversions to farmland appropriately. Sufficient hydrologic conditions provided water protection for agricultural irrigation and plant growth [100,101]. The higher temperature in the south promoted the evaporation of swamps water and provided proper climatic conditions for crop growth in cold temperature regions [23]. Human activity has caused agricultural encroachment into forest swamps for grain production, with numerous promoting policies (e.g., the “Rescinding the Agricultural Tax ”and “Achieving An Additional 50 Million Tons of Grain Production Project”) [23,56], the movement of large populations, and expansion of the agricultural sector in the region [102]. Additionally, studies related to the effects of national policies on swamp reclamation showed that mega projects such as the “One Million Hectare Peat Soil Development Project”, aimed at the development of food crop production, have resulted in the encroachment into swamps in Indonesia [103]. Also, Heimlich and Langner [104] pointed out that agricultural conversion had been the predominant cause of the disappearing forest swamps in the lower Mississippi alluvial plain, with federal flood control and small watershed programs accelerating swamp conversion. However, our study merely considered human interference degree and the distance to roads as the surrogates of the human factor. Selecting more variables related to relevant agricultural policies and population would be more specific and accurate to explore the primary environmental factors for conversions to farmland.

Our study has significant implications for natural resources management and nature conservation. Investigating landscape characteristics in forest swamp changes was warranted as these landscape metrics captured the trend and direction of forest swamp conversions. Exploring the relative importance of environmental factors in forest swamp conversions was conducive to investigating which environmental factors were dominant in different forest swamp conversions. Furthermore, we can conserve and utilize the forest swamp resources reasonably, in accordance with environmental conditions. Additionally, forest swamps can impact the public health through providing water resources, conserving water, homogenizing floods, degrading pollutants, and protecting biodiversity.

## 5. Conclusions

Our study revealed that forest swamping was a common phenomenon. There was a wide distribution occurring at the edge of large areas of forests, meadow swamping was localized with a small amount because of poor water recharge from ground water in the permafrost regions, and water body swamping had complex shape due to the fluctuation of water table and swamp plant growth. We found that geomorphic and climatic factors were common and more important than the topographic factor for forest swamping conversions. Negative geomorphic conditions as a precondition of swamp formation can lead to water to accumulate and form a long-term waterlogging environment. Precipitation was a crucial source of water recharge due to the presence of permafrost layers in cold temperate regions and low temperature not only caused less evaporation but also caused the accumulation rate of the organism to be greater than the decomposition rate. These climatic conditions in cold temperate regions promoted swamp development. Additionally, human activity was a relatively important factor for forest swamping and conversions to farmland. Forest swamping was prone to occurring in burned or logging areas and agricultural reclamation converted swamps into farmland due to grain production and economic pressure. Considering fire disturbance as a variable and selecting more variables related to relevant agricultural policies and population is necessary to further explore the primary and specific environmental factors for forest swamping and conversions to farmland. Due to data limitation, we used distance to rivers as a hydrologic variable rather than the underground water table related to swamps. Thus, more free available data was significant for exploring specific driving variables of forest swamp conversions. Forest swamps play an important role in public health by degrading pollutants, providing water purification, and homogenizing floods. Studying the landscape characteristics and driving mechanisms of forest swamp conversions will be beneficial to the protection and management of forest swamp resources.

## Figures and Tables

**Table 1:** Landscape metrics of forest swamp conversions during 1990–2015.

Forest Swamp Conversions	NP	PD (n/100 ha)	ED (%)	PARA_MN	AI (%)
Forest swamping	34,916	8.51	3.30	313.41	56.66
Meadow swamping	3,613	0.88	2.29	306.26	58.75
Water body swamping	3,791	0.92	0.74	348.32	27.46
Conversion to farmland	4,795	1.17	0.77	289.91	67.82

NP: number of patches; PD: patch density; ED: edge density; PARA_MN: perimeter area ratio mean; AI: aggregation index.

**Table 2:** Environmental factor characterizations for forest swamp conversions.

Factor	Variable	Abbr.	Unit	Characterizations
Topographic	Beer’s aspect	ASP		0.06–2
Topographic	Slope	SLO	°	1.51–42.69
Geomorphic	Geomorphic type	GEO		Mainly includes middle-altitude and medium relief mountains, low-altitude and medium relief mountains, low-altitude and small relief mountains, and low-altitude alluvial plain, alluvial floodplain, and hills.
Climatic	Mean annual precipitation	MAP	mm	374.64–466.71
Climatic	Mean annual temperature	MAT	°C	−4.17–−0.45
Hydrologic	Distance to rivers	RIV	km	The first-order stream and the second-order stream of the study area are selected to make buffers at intervals of 12 km according to the extent of study area.
Edaphic	Soil type	SOI		Mainly includes swampy soil, skeleton soil, meadow soil, dark brown soil, and brown coniferous forest soil.
Human	Distance to roads	ROA	km	Uses the road map to make buffers at intervals of 3 km according to the extent of study area.
Human	Human interference degree	HI		0.02–73.55
